# Serum Proteomic Changes in Pet Rabbits with Subclinical and Clinical Encephalitozoonosis in Thailand

**DOI:** 10.3390/ani15131962

**Published:** 2025-07-03

**Authors:** Taksaon Duangurai, Onrapak Reamtong, Tipparat Thiangtrongjit, Siriluk Jala, Peerut Chienwichai, Naris Thengchaisri

**Affiliations:** 1 Department of Companion Animal Clinical Sciences, Faculty of Veterinary Medicine, Kasetsart University, Bangkok 10900, Thailand; taksaon.du@ku.th; 2 Department of Molecular Tropical Medicine and Genetics, Faculty of Tropical Medicine, Mahidol University, Bangkok 10400, Thailand; onrapak.rea@mahidol.ac.th (O.R.); tipparat.thi@mahidol.ac.th (T.T.); 3 Kamphaeng Saen Veterinary Diagnostic Center, Faculty of Veterinary Medicine, Kasetsart University, Kamphaeng Saen Campus, Nakhon Pathom 73140, Thailand; siriluk.j@ku.th; 4 Princess Srisavangavadhana Faculty of Medicine, Chulabhorn Royal Academy, Bangkok 10210, Thailand; peerut.chi@cra.ac.th; 5 Research Center on Clinical and System Microbiology (RCSyM), Chulabhorn Royal Academy, Bangkok 10210, Thailand

**Keywords:** *Encephalitozoon cuniculi*, gel electrophoresis, mass spectrometry, rabbits, serum proteome

## Abstract

*Encephalitozoon cuniculi* causes both clinical and subclinical infections in rabbits, complicating a diagnosis due to the limitations of conventional tools like ELISA. This study analyzes serum proteomic profiles across clinical, subclinical, and healthy rabbits to identify discriminatory biomarkers. Serum from 90 pet rabbits (30 per group) was pooled (10 samples per pool, 3 pools per group) and analyzed using one-dimensional gel electrophoresis and mass spectrometry. The proteomic analysis revealed 109, 98, and 74 proteins expressed in healthy, subclinical, and clinical groups, respectively. Of these, 50, 40, and 33 proteins were unique to the healthy, subclinical, and clinical groups, respectively, with only 10 proteins shared across all. A total of 88 proteins were differentially expressed in infected groups compared to healthy controls. Importantly, 12 proteins were consistently upregulated in both subclinical and clinical infections. These include markers related to the immune response (beta-2-microglobulin, alpha-2-HS-glycoprotein), coagulation (antithrombin-III, alpha-1-antiproteinase S-1), vitamin A transport (retinol-binding proteins), lipid metabolism (apolipoprotein C-III), cytoskeletal regulation (actin-depolymerizing factor), extracellular matrix integrity (fibrillin 2), and oxidative stress (monooxygenase DBH-like 1). Additionally, Gc-globulin and ER lipid-raft-associated 1 were linked to immune modulation and signaling. These findings identify specific serum proteins as promising biomarkers for distinguishing subclinical from clinical encephalitozoonosis in rabbits, enabling an early diagnosis and effective disease monitoring.

## Simple Summary

*Encephalitozoon cuniculi* is a microsporidian parasite that infects rabbits and other mammals, often affecting the nervous system and sometimes causing anemia, which can complicate a diagnosis, especially in asymptomatic cases. This study analyzed serum proteins in healthy, subclinical, and clinical rabbits using gel electrophoresis and mass spectrometry to identify biomarkers of infection. A total of 88 proteins were differentially expressed in infected rabbits, with 12 consistently upregulated in both subclinical and clinical groups. These proteins are involved in the immune response, coagulation, vitamin A transport, metabolism, and oxidative stress. Key markers included beta-2-microglobulin, antithrombin-III, retinol-binding proteins, and actin-depolymerizing factor. These findings suggest specific blood proteins that may serve as biomarkers for the early detection and clinical differentiation of *E. cuniculi* infection in pet rabbits.

## 1. Introduction

*Encephalitozoon cuniculi*, is an obligate intracellular and spore-forming microsporidian parasite that infects various mammalian hosts, such as rabbits, rodents, dogs, cats, horses, ruminants, primates, and humans, especially those with compromised immune systems [1,2]. *E. cuniculi* primarily infects host cells by extruding its polar filament to inject the infectious sporoplasm, although phagocytosis by host cells has also been reported [3]. Environmental triggers, such as osmotic pressure and pH changes, initiate this process. Inside the host cell, the sporoplasm develops into meronts, which divide into sporonts via merogony [3]. These then mature into spores within a parasitophorous vacuole. Upon rupture of the vacuole, spores disseminate via macrophages or enter the bloodstream [4]. Transmission occurs mainly through the ingestion or inhalation of spores and vertically via transplacental routes [3]. In rabbits, spores typically enter through the intestinal or respiratory tracts, disseminate via infected macrophages, and localize in organs such as the kidneys, liver, lungs, and central nervous system, leading to inflammatory lesions upon host cell rupture [4].

Clinical signs in infected rabbits include neurological issues, such as seizures, paresis, head tilt, ataxia, and circling, along with signs of renal failure, like azotemia, polyuria, polydipsia, and pollakiuria [3,4,5], and ocular lesions of *E. cuniculi* infection mostly present with phacoclastic uveitis, a yellow–white granuloma and a cataract [5,6]. Anemia has been shown to be associated with *E. cuniculi* infection, while leukocytosis, thrombocytopenia, and serum biochemistry showed no links [5]. Transplacental transmission often leads to intraocular infection in young rabbits and causes uveitis [7]. However, the clinical severity varies with the immune status, with immunosuppressed rabbits experiencing severe clinical signs and high mortality [4]. While a significant portion of infected individuals remain subclinical, accurate detection is crucial, necessitating specialized staining or electron microscopy in laboratory diagnostics. The presence of subclinical *E. cuniculi* infections without obvious clinical signs suggests that the disease may be more widespread than previously recognized. Serological testing is one of the most widely used techniques to detect the *E. cuniculi* antibody titer in alive rabbits [8]. However, this method is quite limited [5,8]. A positive result only indicates that the animals have previous exposure *to E. cuniculi*, with no exact information as to when this occurred [9]. Clinical signs are not certainly associated with a high antibody titer [5,9]. A negative result of the antibody titer can rule out encephalitozoonosis [5,9]. In addition, DNA-based methods, by detecting samples from feces, urine, and cerebrospinal fluid, have been reported to have low sensitivity, which can lead to false negative results [8]. In contrast to other diagnostic methods, proteomic studies can be used to examine protein patterns in both clinical and subclinical *E. cuniculi* infections, compared to healthy rabbits. This approach offers valuable insights into the pathogenesis of *E. cuniculi* and could enhance the diagnostic accuracy.

The study aimed to identify and compare the differential protein expression in the sera of healthy, subclinical, and clinical *E. cuniculi*-infected pet rabbits using a proteomic analysis. The study sought to characterize the functional roles of these proteins in the immune response, coagulation cascades, inflammation, and cellular processes. Additionally, it aimed to identify potential biomarkers for subclinical and clinical encephalitozoonosis, providing insights into host–pathogen interactions and disease progression in pet rabbits.

## 2. Materials and Methods

### 2.1. Study Population

Rabbit sera were obtained from our previous study, which is described in detail in a prior report [5]. It included a total of 90 pet rabbits at the time of their first visit to the Kasetsart University Veterinary Teaching Hospital, Faculty of Veterinary Medicine, Kasetsart University (Bangkok, Thailand) between 2020 and 2022. Prior to sample collection, owners provided informed consent by signing consent forms. Specifically, this study categorizes the rabbits based on Duangurai et al. (2024) [5], and the rabbits were divided into three groups according to their serological status and clinical presentation: Group 1 (healthy; *N* = 30): rabbits seronegative for anti-*E. cuniculi* antibodies via ELISA (*N* = 30). Group 2 (subclinical; *N* = 30): rabbits seropositive for anti-*E. cuniculi* antibodies but did not display overt clinical signs at the time of evaluation were presented for routine wellness examinations, assessments of unrelated clinical conditions, or as part of broader health screening protocols. Group 3 (clinical; *N* = 30): rabbits seropositive for anti-*E. cuniculi* antibodies with one or more clinical signs, such as vestibular disease (e.g., head tilt, nystagmus), urinary disorders (e.g., polyuria, urine scalding), ocular abnormalities (e.g., uveitis, cataracts), or gastrointestinal issues (e.g., hypomotility, anorexia). Neurological signs were the most prevalent, with head tilt, ataxia, and seizures commonly observed. The duration of clinical signs varied, with both acute and chronic presentations, depending on the time of clinical sign recognition by the owner and subsequent veterinary consultation. Differential diagnoses, particularly otitis interna and other causes of neurological or renal diseases, were carefully evaluated and excluded through comprehensive clinical examinations, diagnostic imaging, and laboratory testing.

### 2.2. Preparation of Rabbit Sera

All animal procedures were approved by the Kasetsart University institutional Animal Care and Use Committee (ACKU64-VET-023, License No. U1-00723-2558). The pet rabbit blood used in this study was collected from the saphenous vein. Approximately 1000 μL of blood was collected and allowed to clot by leaving the tube undisturbed for 30 min at room temperature. Serum was subsequently obtained via centrifugation at 2000× *g* for 10 min at 4 °C and stored at −20 °C until further use. Three technical replicates were obtained. Rabbit sera were analyzed for the protein profiles of each rabbit group.

### 2.3. Serum Protein Separation

**Figure 1:**
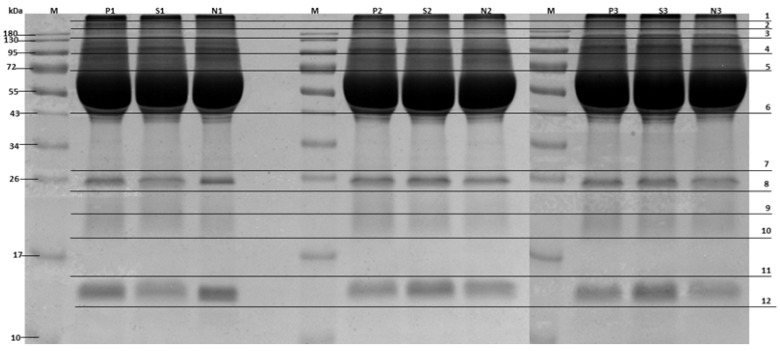
SDS-PAGE of *E. cuniculi*: clinical, subclinical, and healthy rabbit sera. Lanes (from left to right): marker (M), clinical (P), subclinical (S), and healthy (N) rabbit sample. Each group was conducted in triplicate (no. 1–3). The 12 horizontal sections show the regions excised for mass spectrometry analyses.

To identify changes in the concentration of serum proteins between rabbit groups. Serum samples were collected from rabbits categorized into three groups: healthy, subclinical, and clinical (*N* = 30 rabbits per group). To minimize biological variability and ensure a sufficient sample volume for proteomic analysis, samples within each group were pooled. Specifically, ten individual serum samples were combined to create one pooled sample, resulting in three pooled samples per group (3 pooled samples × 3 groups = 9 pooled samples in total). Each of these pooled samples was then subjected to mass spectrometric analysis in technical triplicate to ensure reproducibility and data reliability. A 30 μg aliquot of pooled serum from each group was separated using 12% SDS-PAGE and subsequently analyzed via mass spectrometry. Protein bands were visualized through staining with Coomassie Blue G, excised from the gel, and cut into 12 small pieces for in-gel digestion (Figure 1).

### 2.4. In-Gel Digestion

Coomassie dye was removed by incubating the gels in 25 mM ammonium bicarbonate buffer containing 50% acetonitrile. Proteins were reduced with 4 mM dithiothreitol (Sigma-Aldrich, St. Louis, MO, USA) in 50 mM ammonium bicarbonate buffer, then alkylated with 250 mM iodoacetamide (Sigma-Aldrich, St. Louis, MO, USA), and dehydrated with 100% acetonitrile. After removing the supernatant, proteins were digested overnight with 10 ng trypsin (Sigma Aldrich, St. Louis, MO, USA) dissolved in 200 μL of 50 mM ammonium bicarbonate buffer containing 5% acetonitrile. The peptides were extracted by adding 200 μL of acetonitrile and incubating for 20 min. The supernatant containing the peptides was transferred to a new tube and dried using a centrifugal vacuum concentrator and then dissolved in 0.1% *v*/*v* formic acid.

### 2.5. Mass Spectrometric Analysis

A mixture of tryptic peptides was analyzed using a nano-liquid chromatography system (Dionex UltiMate 3000, Surrey, UK) coupled to a micro TOF-Q mass spectrometer (Bruker Daltonics, Bremen, Germany). Peptide separation was achieved on an Acclaim PepMap RSLC nanoviper C18 column (75 μm × 15 cm, 2 μm particle size, 100 Å pore size; Thermo Scientific, Waltham, MA, USA) at a flow rate of 300 nL/min. A 30 min linear gradient elution was applied from 4% to 50% solvent B (typically acetonitrile with 0.1% formic acid). Mass spectrometric analysis was performed using electrospray ionization (ESI) in positive ion mode. MS and MS/MS data were acquired in data-dependent acquisition mode. Full MS scans were acquired in the *m*/*z* range of 400–2000, and MS/MS scans covered 50–1500 *m*/*z*. The capillary voltage was set to 4.5 kV, with a source temperature of 150 °C. External calibration was performed daily using a standard peptide mixture to ensure mass accuracy within 5 ppm. Lock mass correction was applied where available.

The peptide mass spectra were processed using DataAnalysis 3.4 (Bruker Daltonics), and Mascot generic files (.mgf) were generated for identification. The .mgf files were merged and analyzed using Mascot Daemon version 2.3.2 (Matrix Science, London, UK). The search parameters included a maximum of one missed tryptic cleavage, a peptide mass tolerance of 0.8 Da, and a fragment mass tolerance of 0.8 Da. Variable modifications included methionine oxidation and cysteine carbamidomethylation. Protein identification was performed using the *E. cuniculi* protein database from the National Center for Biotechnology Information (NCBI). The Mascot search results were filtered to achieve a false discovery rate (FDR) of <5% at the peptide level. Proteins were considered reliably identified if at least two unique peptides were detected per protein.

Protein abundance was semi-quantitatively estimated using the exponentially modified Protein Abundance Index (emPAI). Significance in protein abundance was determined based on fold changes in spectral counts between subclinical vs. healthy and clinical vs. healthy groups. A protein was considered differentially abundant if the fold change was >1.0 or <0.5.

### 2.6. Bioinformatics

All identified proteins were classified based on their functions using the UniProt database, release 2025_01. Subsequently, the Venn diagram [10] was created to compare the functions of proteins across all groups. Protein–protein interactions were analyzed using the STRING database Available online: (https://string-db.org/) (accessed on 15 February 2025). This analysis was conducted by restricting the species to “*Oryctolagus cuniculus*” and setting the minimum required interaction score to a medium confidence > 0.4.

### 2.7. Statistical Analysis

This study established the following criteria: a twofold (fold change) increase or decrease in protein expression levels, and the presence of the protein in at least two out of three samples within one of the study groups, in accordance with the criteria for the selected protein expression set in previous research [11]. Protein sequences were analyzed using BLAST2GO and the KEGG Automatic Annotation Server (KAAS). Additionally, protein sequences were mapped to KEGG pathways using standard parameters. The top 20 annotated pathways were compiled, with a focus on those related to clinical encephalitozoonosis, and the identified proteins were clearly marked.

## 3. Results

**Figure 2:**
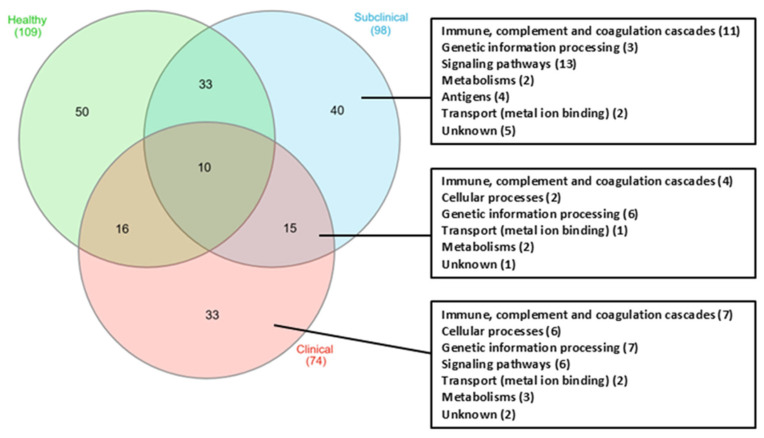
Venn diagram showing the number of expressed proteins identified in healthy (green), subclinical (blue), and clinical rabbit groups (red). The protein biological pathways of the overlapping section of both subclinical and clinical groups, proteins only identified in subclinical, and proteins only identified in clinical groups are listed on the right.

Differential protein concentrations in rabbit sera from healthy, subclinical, and clinical groups were analyzed using SDS-PAGE (Figure 1) and mass spectrometry. A comparison of protein expression across groups revealed 109, 98, and 74 proteins in the healthy, subclinical, and clinical groups, respectively (Figure 2). Of these, 50 proteins were exclusive to the healthy group, 40 to the subclinical group, and 33 to the clinical group. Ten proteins were commonly expressed across all groups, while 88 proteins exhibited differential expression in the subclinical and clinical groups compared to the healthy group. Notably, 15 proteins were found exclusively in the subclinical and clinical groups. Among these, only 12 represented unique protein types with a fold change in emPAI greater than 2. 

The top 20 annotated pathways, with a focus on clinical encephalitozoonosis, are presented with identified proteins marked. Table 1 outlines the differential protein expression in healthy, subclinical, and clinical *E. cuniculi* groups. These proteins were chosen based on the magnitude of their fold change and statistical significance, as determined via LC-MS/MS quantification using emPAI values. This table exclusively presents the top 20 differentially expressed proteins within each comparative group (healthy vs. subclinical, healthy vs. clinical, and subclinical vs. clinical). The aim was to highlight the most prominent and statistically robust changes in protein abundance, representing the strongest proteomic alterations specific to each disease stage. Protein changes indicate upregulation in both clinical and subclinical groups, being absent in healthy rabbits, suggesting immune activation or pathogen-driven immune evasion.

**Figure 3:**
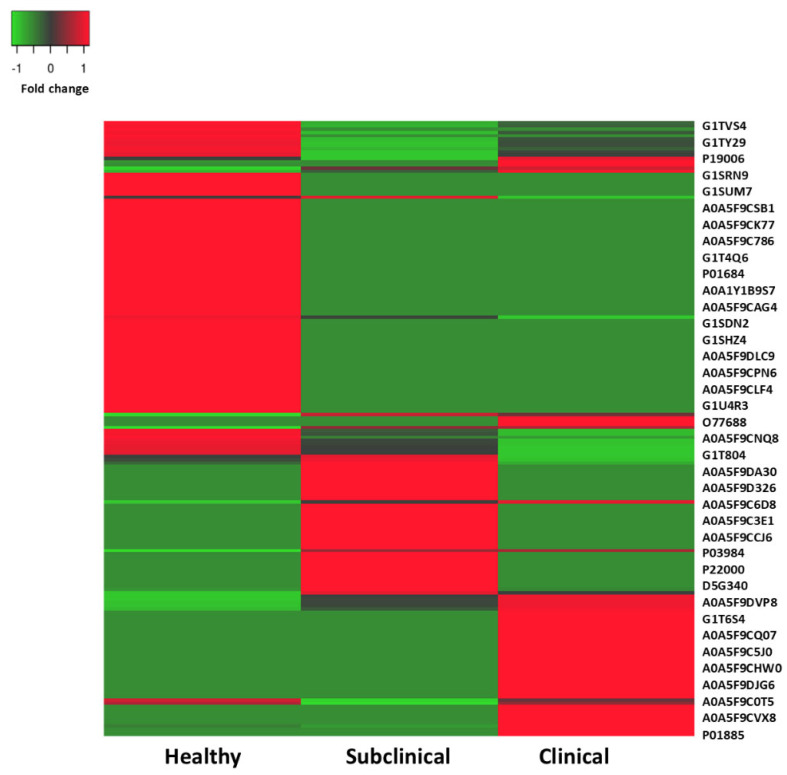
Heatmap showing the up− and downregulated proteins. The vertical axis represents the Uniport ID, which was used to compare their expression levels among the rabbit groups (healthy, subclinical, and clinical). Red and green represent the up− and downregulated proteins, respectively. The dark red color in the figure indicates the upregulated proteins expressed in both the Subclinical and Clinical groups, while the red color represents proteins that are upregulated exclusively in the Subclinical or Clinical group, respectively. These proteins are of particular interest in this study. The *x*–axis represents the protein accession numbers, while the *y*–axis shows the fold change in expression across the study groups.

A broader spectrum of proteins that exhibited statistically significant differential expression levels across all three clinical categories (healthy, subclinical, and clinical) is shown in Figure 3. This heatmap visualization highlights comprehensive expression patterns, including upregulation, downregulation, and consistent trends based on the fold change. Proteins were included in Figure 3 if their expression patterns were biologically relevant. This includes those that were uniquely or consistently regulated in the subclinical and/or clinical groups, even if they did not meet the stringent top 20-fold change criteria used for Table 1. This approach provides a more holistic view of the proteomic shifts throughout the infection stages. A protein expression heatmap, which is a graphical representation used to visualize the expression levels (fold change) of different proteins across all groups, is presented in Figure 3, where red and green represent upregulated (red) and downregulated (green) proteins, respectively. The dark red color in Figure 3 indicates the upregulated proteins expressed in both the subclinical and clinical groups, while the lighter red color represents proteins that are upregulated exclusively in either the subclinical or clinical group. The *x*-axis represents the protein accession numbers, while the *y*-axis shows the fold change in expression across the study groups. These proteins are of particular interest in this study. The findings reveal a range of proteomic functions involved in the host immune response, inflammatory response, coagulation cascades, and cellular processes in the subclinical and clinical groups compared to healthy controls. Notably, proteins such as prothrombin, antithrombin, alpha-2-macroglobulin, beta-2-microglobulin, beta-1 metal-binding globulin, and complement component 4-binding protein alpha were identified as playing roles in the immune system and coagulation cascade. Furthermore, the expression of haptoglobin, phosphodiesterase 4D interacting protein, alpha-2-HS-glycoprotein, and protein AMBP may be associated with inflammatory conditions. Interestingly, *E. cuniculi*-derived proteins were also identified, including actin-depolymerizing factor and retinol-binding protein, both of which were over-expressed in the subclinical and clinical groups.

**Figure 4:**
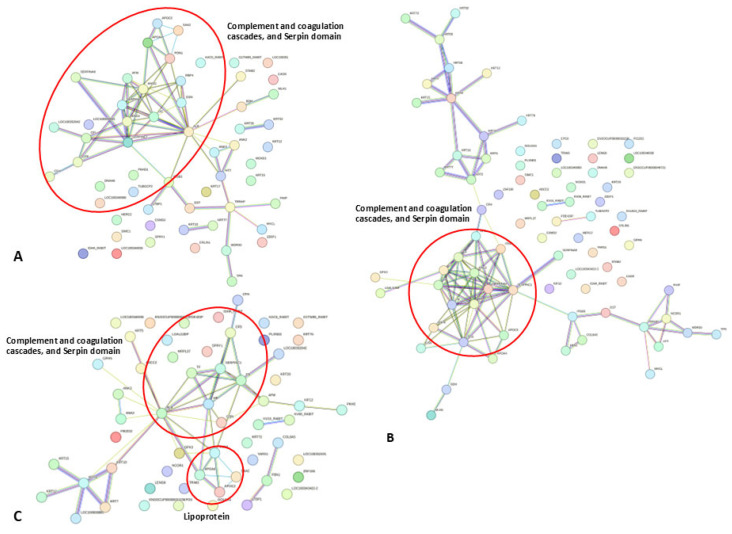
Diagrams revealing protein–protein interaction networks of differentially expressed proteins in *E. cuniculi*-infected rabbits. (**A**) The protein–protein interactions of serum proteins only identified in subclinical and clinical *E. cuniculi* rabbit groups. (**B**) The protein–protein interactions of serum proteins only identified in clinical rabbit groups. (**C**) The protein–protein interactions of serum proteins only identified in subclinical rabbit groups. Node colors represent pathway or functional enrichment categories as assigned by STRING. Proteins outlined in red circles indicate key pathway-associated proteins within the interaction network.

The prediction of protein–protein interaction networks of the expressed proteins, protein ID lists from rabbit serum, was submitted to STRING analysis (Figure 4A–C). The resulting interaction networks revealed molecular processes, including complement and coagulation pathways, associated with *E. cuniculi* infection in subclinical and clinical groups (Figure 4A). The predicted interactions of proteins exclusively expressed in clinical and subclinical groups are shown in Figure 4B and Figure 4C, respectively. This analysis indicates a strong interaction between coagulation pathways and the immune response, providing additional insight into host responses during encephalitozoonosis.

## 4. Discussion

Our study identified distinct proteomic profiles in subclinical and clinical rabbits compared to healthy controls, suggesting potential biomarkers and providing insights into the host’s response to *E. cuniculi* infection. The upregulated proteins were associated with *E. cuniculi*, including those related to the immune system, complement and coagulation cascades, cellular processes, genetic information processing, signaling pathways, metal ion transport, and metabolism. Both innate and adaptive immune responses play essential roles in the host’s defense against microsporidian infections, contributing significantly to the development of resistance and partial control of the pathogen [12]. Upon infection, several immune cell types, including macrophages, dendritic cells, and CD8^+^ T lymphocytes are activated to recognize and eliminate the parasite [12]. In addition, key cytokines such as interleukin-12 (IL-12) and interferon-gamma (IFN-γ) have been shown to orchestrate these immune processes by enhancing cellular and humoral immunity [12]. Nevertheless, these immune mechanisms are generally insufficient to achieve complete eradication of the parasite. *E. cuniculi*, in particular, has evolved strategies to subvert host innate immune defenses, manipulating immune signaling pathways to ensure its persistence within host tissues [13]. This finding is consistent with previous reports and our study indicating that all infected animals exhibit elevated IgG levels, suggesting a robust humoral immune response [5,14]. However, the presence of elevated IgG titers is not necessarily indicative of active or severe infection, as rabbits with prior exposure or recovered infections may also maintain high levels of circulating IgG [5,14]. Furthermore, a proteomic analysis showed that complement and immunoglobulin components were present only in subclinical and clinical rabbits, indicating their role in immune defense against *E. cuniculi* and suggesting the complexity of host responses.

A previous study provided insights into the clinical and serological features of *E. cuniculi* infection in pet rabbits [5]. The study has shown that *E. cuniculi* affects multiple organ systems, with pet rabbits being particularly susceptible to anemia and neurological signs, while traditional diagnostic methods like ELISA cannot differentiate between clinical and subclinical cases [5]. Our subsequent study aimed to identify a proteomic analysis to distinguish among clinical, subclinical *E. cuniculi*, and healthy rabbit groups. In this study, *E. cuniculi* infection induced significant alterations in protein expression, reflecting the progression of the disease. In the clinical phase of infection, proteins such as prothrombin (fragment), complement component 4 binding protein alpha, and alpha-2-macroglobulin are upregulated, indicating an immune response. While this immune activation is crucial for combating the pathogen, it can also contribute to the disease pathology, especially if immune mechanisms fail to control the infection. Additionally, the upregulation of proteins like antithrombin-III and sprouty homolog 1 protein suggests disruptions in normal immune and metabolic functions, which may facilitate the onset of clinical signs. The overexpression of antithrombin-III, as seen in leptospirosis, suggests that coagulation pathway activation may contribute to anemia and multi-organ failure in encephalitozoonosis [15]. Although no direct interaction between *E. cuniculi* and antithrombin has been demonstrated, the evidence supports the hypothesis that indirect mechanisms of coagulation activation significantly contribute to the severe clinical manifestations observed in encephalitozoonosis. Additionally, sprouty protein, a known negative regulator of receptor tyrosine kinase signaling, particularly the mitogen-activated protein kinase/extracellular signal-regulated kinase (MAPK/ERK) pathways, plays an essential role in modulating immune responses and inflammatory processes [16]. These pathways are integral to cell proliferation, survival, immune cell activation, and the regulation of inflammatory responses [16]. The sprouty protein functions by attenuating immune signaling cascades, thereby preventing excessive inflammation during infectious processes [17]. To date, however, no direct evidence has been reported linking the sprouty protein to *E. cuniculi* infection.

Proteins like retinol-binding protein 4 and plasma retinol-binding protein were specifically elevated in the clinical group, potentially indicating organ damage and the severity of the disease, serving as markers for tissue damage in clinical cases. *E. cuniculi* infection triggers the activation of both the inflammatory response and coagulation cascades [18], contributing to tissue damage and vascular injury [19] in affected animals in various visceral organs, such as the brain, kidneys, heart, and liver in infected rabbits [20]. In this study, proteins such as prothrombin, alpha-2-macroglobulin, and haptoglobin, which are involved in coagulation and inflammation, showed increased expression in subclinical and clinical cases. Prothrombin, essential for blood clotting, may be upregulated in response to tissue injury and inflammation caused by *E. cuniculi* infection, potentially contributing to vascular thrombosis and exacerbating tissue damage [21,22]. Prothrombin’s role in clot formation may help assess thrombotic risks [23], while alpha-2-macroglobulin could be targeted to reduce excessive clot formation and tissue damage [24,25]. An increase in blood prothrombin concentrations has been reported in Huntington’s disease, a progressive neurodegenerative disease in humans [26]. Additionally, chronic anemia, which is commonly associated with *E. cuniculi* infection, may indirectly activate inflammatory and procoagulant pathways, further influencing clot formation and immune responses [5].

In subclinical *E. cuniculi* infection, proteins such as Gc-globulin and alpha-2-HS-glycoprotein show increased expression, suggesting they may play a protective role in preventing the disease from progressing into more severe clinical signs. These proteins likely help mitigate the infection’s effects, assisting the host in maintaining homeostasis and avoiding overt clinical signs. In contrast, the clinical group showed the downregulation of proteins like albumin and serum amyloid A-2 protein, which are crucial for maintaining cellular processes, indicating disruptions in normal physiological functions. Additionally, proteins like Uncharacterized protein (38,221) and Uncharacterized protein (70,533) were downregulated in subclinical cases, possibly reflecting compensatory mechanisms aimed at maintaining a balance. However, these mechanisms become less necessary as the infection progresses into the clinical stage. Proteins such as alpha-2-macroglobulin, expressed in both subclinical and clinical groups, play a key role in regulating clot formation, modulating inflammatory processes by inhibiting proteases regulating of inflammation [27] and immune responses [24], thereby preventing excessive clot breakdown during infection and protecting against tissue damage [24,25,27]. Haptoglobin, an acute-phase protein, also showed increased expression, indicating its role in tissue protection and limiting clotting activation [28,29]. Furthermore, both haptoglobin and alpha-2-macroglobulin were previously reported to be overexpressed in *E. cuniculi*-infected rabbits exhibiting neurological signs [30]. This is consistent with our findings; however, it remains unclear why this protein was also present in the subclinical group. The antithrombin overexpression in both groups suggests a shift in the coagulation regulation, which could influence thrombotic and immune responses during *E. cuniculi* infection. Further studies are needed to clarify the complex interactions among these proteins, immune responses, and the pathophysiology of the infection. Cytoskeletal and cellular remodeling plays a critical role in the pathogenesis of *E. cuniculi*, particularly in host cell invasion and immune evasion. One key factor involved in this process is the actin-depolymerizing factor (ADF), which facilitates the motility of the pathogen and host cell invasion by disrupting the actin cytoskeleton. While the role of ADF in *E. cuniculi* pathogenesis remains poorly understood, it shares similarities with other pathogens, such as *Toxoplasma gondii* and *Cryptosporidium parvum*, in utilizing host microfilaments for invasion [31,32].

**Figure 5:**
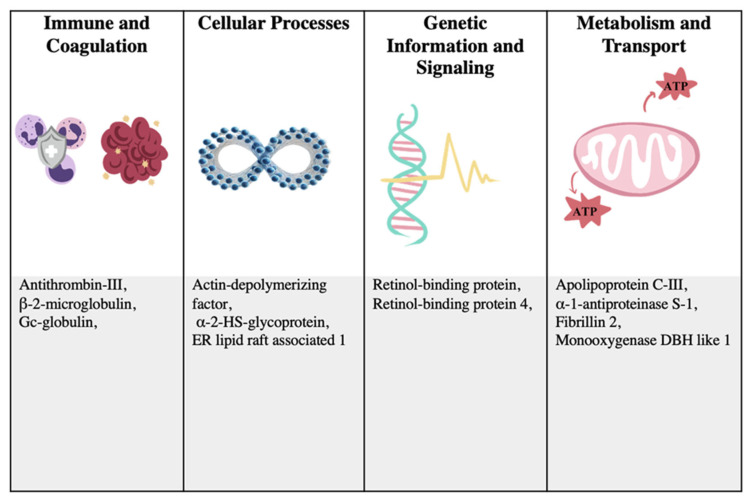
The key proteomic changes in rabbits with active *E. cuniculi* infection, emphasizing the complex interactions among inflammation, coagulation, and immune responses with significant clinical implications (fold change greater than twofold).

The key proteomic changes in rabbits with active *E. cuniculi* infection are summarized in Figure 5, demonstrating the complex interplay among inflammation, coagulation, and immune responses, which have significant clinical implications. The overexpression of antithrombin, a coagulation-related protein, suggests its potential as a biomarker for early detection, disease monitoring, and therapeutic targeting. Additionally, the finding of proteins involved in the immune response (beta-2-microglobulin, alpha-2-HS-glycoprotein), inflammatory response (alpha-1-antiproteinase S-1), vitamin A transport (retinol-binding proteins), lipid metabolism (apolipoprotein C-III), cytoskeletal changes (actin-depolymerizing factor), extracellular matrix structure (fibrillin 2), and oxidative stress (monooxygenase DBH-like 1), with Gc-globulin and ER lipid raft associated 1 implicated in immune modulation and signaling, suggests that these proteins may contribute to a tightly regulated immune environment [33,34,35]. This modulation likely facilitates a subclinical phase of infection, allowing the host to maintain immune homeostasis and delay the onset of clinical signs despite the presence of the pathogen. The excessive inflammation observed during encephalitozoonosis in rabbits may be modulated by elevated levels of host serine protease inhibitors, particularly alpha-1-antiproteinase. This serpin family enzyme is known to play a critical role in regulating inflammatory responses. Previous studies have reported the overexpression of alpha-1-antiproteinase in rabbits infected with *E. cuniculi*, suggesting its potential involvement in controlling inflammation during infection [30]. Moreover, high levels of proinflammatory cytokines during inflammation, such as TNF and IFN-γ, play a critical role in exacerbating disease severity. In response to this immunological imbalance, the liver activates a counter-regulatory pathway by synthesizing acute phase proteins, notably alpha-2-HS-glycoprotein. Alpha-2-HS-glycoprotein has been recognized for its neuroprotective capacity and its potent anti-inflammatory properties, particularly in the context of systemic inflammatory conditions, such as sepsis and autoimmune disorders [36,37]. The identification of neuroinflammation-related protein like retinol-binding protein points to potential biomarkers for neurological damage, particularly in the early stages of encephalitozoonosis [38]. These markers linked to neurodegenerative diseases may help diagnose brain involvement and predict complications.

This study identified distinct proteomic differences between the subclinical and clinical phases of infection, underscoring their potential utility as diagnostic biomarkers. Several immune-related proteins, including haptoglobin and alpha-2-macroglobulin, were elevated in both infected groups. However, specific proteins exhibited phase-dependent expression patterns. Notably, Gc-globulin and alpha-2-HS-glycoprotein were predominantly upregulated in the subclinical group, suggesting a protective or modulatory function. These proteins may contribute to the maintenance of homeostasis and the immune balance, potentially delaying or preventing progression to clinical disease. Their expression profiles suggest their utility as early indicators of infection, prior to the onset of overt clinical signs. In contrast, proteins such as retinol-binding protein 4, plasma retinol-binding protein, and sprouty homolog 1 protein were either exclusively or markedly elevated in the clinical group. These proteins are associated with organ damage, inflammation, and signal transduction dysregulation, and they may serve as indicators of disease severity and systemic involvement. Additionally, the downregulation of albumin and serum amyloid A-2 in clinical cases, compared to subclinical cases, suggests a transition towards impaired physiological function and inflammation-induced tissue injury. This proteomic shift further underscores the progressive nature of the host–pathogen interaction and emphasizes the importance of identifying early-phase biomarkers. Furthermore, several proteins demonstrated expression changes associated with infection, regardless of the disease phase. For example, antithrombin-III was upregulated in both the subclinical and clinical groups, potentially reflecting a generalized response to infection or coagulation pathway activation. Similarly, complement component 4-binding protein alpha and prothrombin fragment levels were elevated in infected animals, indicating involvement in immune response regulation. Collectively, these findings support the development of a biomarker panel capable of differentiating between the subclinical and clinical stages of infection. Early markers, such as Gc-globulin and alpha-2-HS-glycoprotein, may facilitate the detection of latent infections; the infected individual carries the pathogen but does not exhibit overt clinical signs. The identification of specific proteomic signatures in the subclinical phase provides a means to detect these hidden infections, which is critical for disease control and management, particularly in susceptible populations. While biomarkers such as retinol-binding proteins, SPRY1, and antithrombin-III could serve as indicators of advanced disease. This approach has practical implications for early diagnosis, disease monitoring, and longitudinal surveillance, particularly in high-risk populations such as breeding colonies and laboratory rabbits.

While our proteomic study offers valuable insights into *E. cuniculi* infection, it has several limitations. The functional roles of identified proteins in rabbits remain poorly understood. Our small sample size may limit generalizability; larger, more diverse cohorts are needed for a robust results. Furthermore, it is important to acknowledge the use of pooled samples in this study. Each pooled sample represented sera from 10 individual rabbits, with three independent pools created per group. While this approach aimed to reduce biological variability and detect consistent group-level trends, it inherently limits the analysis of individual-level variability. Future studies could explore non-pooled samples to capture individual responses more comprehensively. The heterogeneity within the clinical group, influenced by variables such as age and the infection duration, may have affected proteomic profiles and immune marker expression, warranting a stratified subgroup analysis in future research. Moreover, the analysis targeted a limited subset of proteins, possibly overlooking additional relevant biomarkers, and the cross-sectional study design restricts an understanding of the dynamic protein expression over time. Longitudinal studies are needed to clarify causal relationships and the roles of these proteins in disease progression. Functional validation using specific antibodies will be essential to translate these findings into accurate diagnostic tools and targeted treatments. An additional limitation is the presence of unrelated clinical conditions in some subclinical-group animals, which may have confounded the proteomic profiles, particularly immune and inflammatory markers, by introducing non-specific responses that obscure infection-specific signals. To improve the specificity and reliability of biomarker identification, future studies must adopt stricter inclusion criteria and use better-matched control groups. Furthermore, the mechanisms driving the reactivation of latent *E. cuniculi* infection in immunocompromised rabbits, often resulting in severe encephalitozoonosis, remain poorly understood and warrant further investigations.

## 5. Conclusions

The present study identified 281 total serum proteins across healthy, subclinical, and clinical rabbit groups, with 88 proteins differentially expressed in clinical infection groups compared to healthy controls. Notably, 12 proteins were consistently present in both subclinical and clinical cases, shedding light on the key molecular changes associated with *Encephalitozoon cuniculi* infection. This proteomic analysis offers critical insights into the biological pathways involved in coagulation, inflammation, neurodegeneration, immune evasion, and kidney injury. These findings identify distinct serum proteins as promising biomarkers with strong potential to accurately differentiate subclinical from clinical encephalitozoonosis in rabbits, offering a powerful tool for early diagnosis and effective disease monitoring. The findings also enhance our understanding of disease pathogenesis and may aid in developing diagnosis tools, predicting outcomes, and creating targeted therapeutic interventions. Further validation through larger cohorts, functional analyses, and longitudinal studies is needed to confirm their clinical value.

## Figures and Tables

**Table 1:** Protein patterns of clinical, subclinical, and healthy Groups. A combination of the Top-20 differential rabbit serum proteins from pairwise comparisons: clinical vs. healthy, subclinical vs. heathy, and clinical vs. subclinical in rabbits with *E. cuniculi* infections. Upregulated and downregulated proteins, determined by comparing serum proteins using LC-MS/MS and the UniProt protein database, were identified in this comparison. The fold change between two groups was calculated as, for example, the mean emPAI (Clin)/mean emPAI (Healthy).

Access No.	Protein Name	M.W.	No. of Peptide	Fold Change (Clinical vs. Healthy)	Fold Change (Subclinical vs. Healthy)	Fold Change (Clinical vs. Subclinical)
Immune, complement and coagulation cascades						
O77688	Prothrombin (Fragment)	12,161	1	ON *	ON *	1.08
A0A5F9CCJ6	Complement component 4 binding protein alpha	65,840	3	ON *	ON *	1.17
A0A5F9CNX4	Alpha-2-macroglobulin	156,458	11	ON *	ON *	1.05
G1SIK0	Antithrombin-III	52,603	4	4.33	2	2.15
P19006	Haptoglobin	36,434	2	4.25	3.25	1.3
P01885	Beta-2-microglobulin	11,647	2	ON *	—	ON *
G1SU82	Gc-globulin	53,913	2	ON *	—	ON *
G1TW85	Ig-like domain-containing protein	11,641	12	0.73	OFF **	ON *
G1SS69	C3/C5 convertase	85,298	3	OFF **	0.5	OFF **
A0A1Y1B9L0	IgG light chain (Fragment)	22,862	5	—	ON **	OFF **
A0A0C6G056	IgM light chain	22,428	3	—	ON *	OFF **
P03988	Ig mu chain C region secreted form	49,866	9	—	ON *	OFF **
A0A0C6G052	IgG heavy chain VDJ region	23,629	3	—	ON *	OFF **
P03984	Ig kappa chain b5 variant C region	11,072	2	—	ON *	OFF **
P22000	Serum amyloid A-2 protein	13,443	5	—	ON *	OFF **
Cellular processes						
G1U9R8	Actin-depolymerizing factor	81,346	7	ON *	ON *	2.82
U3KMR2	Alpha-2-HS-glycoprotein	36,005	6	ON *	ON *	2.15
G1TIT1	ER lipid raft associated 1	39,027	4	ON *	—	ON*
Genetic information processing						
A0A5F9DVP8	Retinol-binding protein	24,043	10	ON *	ON *	2.29
P04916	Retinol-binding protein	23,205	5	ON *	ON *	2.29
P06912	Retinol-binding protein 4	23,087	11	2.7	1	2.7
G1SZH0	Plasma retinol-binding protein	49,814	13	2.38	1.06	2.26
A0A5F9C9K8	Phosphodiesterase 4D interacting protein	278,239	10	ON *	ON *	1.0
A0A5F9C619	Remodeling and spacing factor 1	158,986	5	ON *	ON *	1.0
G1T8D0	Sprouty homolog 1 protein	34,904	4	1	2	0.5
Transport (metal ion binding)						
G1STF7	Beta-1 metal-binding globulin	76,635	39	ON *	ON *	0.77
A0A5F9DMU6	Inter-alpha-trypsin inhibitor heavy chain H3	98,412	5	0.28	0.5	0.5
G1SV85	Monooxygenase DBH like 1	71,508	5	ON *	—	ON *
P49065	Albumin	68,865	44	—	ON *	OFF *
Signaling pathways						
A0A5F9CQ07	Fibrillin 2	311,658	21	ON *	—	ON *
P27170	Serum paraoxonase/aryl- esterase 1	39,984	5	—	ON *	OFF *
Metabolism						
A0A5F9CSE7	Protein AMBP	49,014	3	ON *	ON *	1.5
B7NZM1	Apolipoprotein A-I (Predicted)	30,585	26	—	ON *	OFF **
G1U2V8	Apolipoprotein C-III	17,367	1	ON *	—	ON *
Q07298	Alpha-1-antiproteinase S-1	45,721	4	ON *	—	ON *
Unknown						
G1U6P5	Uncharacterized protein	38,221	4	0.44	0.21	2.07
A0A5F9C4L3	Uncharacterized protein	70,533	7	ON *	—	ON *
A0A5F9D345	Uncharacterized protein	54,050	7	OFF **	2	OFF **
G1T804	Uncharacterized protein	34,065	2	OFF **	0.21	OFF **
G1TBS8	Uncharacterized protein	139,280	31	OFF **	3.71	OFF **

Notes: ON (clinical) means that the protein was only detected in the group. OFF (subclinical) means the protein was not detected in the group, — means the protein was not detected via MS (undefined). * *p* < 0.05, **p < 0.01 for pairwise comparisons.

## Data Availability

The datasets generated and/or analyzed during the current study are available from the corresponding author upon reasonable request.
